# Adherence to medical recommendations in high-risk pregnancy: dispositional and situational predictors with a focus on emotional reactivity

**DOI:** 10.1017/S1092852925100436

**Published:** 2025-07-21

**Authors:** Manuel Glauco Carbone, Concetta Polizzi, Maria Maddalena Di Pasqua, Maria Regina Morales, Giovanna Perricone, Gaspare Cucinella, Rosalia Sutera, Sofia Burgio, Giulia Giordano

**Affiliations:** 1 Division of Psychiatry, Department of Medicine and Surgery, University of Insubria, Varese, Italy; 2 Saint Camillus International University of Health Sciences, Rome, Italy; 3 Department of Psychology, Educational Science, and Human Movement (SPPEFF), University of Palermo, Palermo, Italy; 4 Italian Society of Pediatric Psychology (S.I.P.Ped), Rome, Italy; 5 Comune di Palermo, Garante dell’Infanzia e dell’Adolescenza, Palermo, Italy; 6 Division of Obstetrics and Gynaecology, V. Cervello Hospital, University of Palermo, Palermo, Italy; 7 Maternal and Child Health Department, V. Cervello Hospital, Palermo, Italy

**Keywords:** medication adherence, high-risk pregnancy, personality traits, sensory processing sensitivity, pregnancy intention

## Abstract

**Objective:**

Therapeutic adherence during pregnancy is critical for maternal and fetal health. This study examines personality traits, sensitivity to stimuli and socio-demographic factors influencing adherence among Italian women with high-risk pregnancies.

**Methods:**

Ninety women from “Villa Sofia—V. Cervello Hospital”, in Palermo, Italy, participated. Personality traits were assessed via the Personality Inventory (PI), covering Extraversion, Conscientiousness, Neuroticism, Mental Openness, and Friendliness. Sensitivity to stimuli was evaluated using the Highly Sensitive Person (HSP) Scale, which includes Low Sensory Threshold (LST), Ease of Excitement (EOE), and Aesthetic Sensitivity (AES). Treatment adherence was measured using the Morisky Medication Adherence Scale (MMAS).

**Results:**

Conscientiousness was identified as a positive predictor of medication adherence (OR = 1.08, p = .010), while Mental Openness (OR = 0.81, p = .003) and EOE (OR = 0.92, p = .014) were negative predictors. Higher education levels were associated with better adherence (OR = 2.34, p = .006). Significant occupational differences emerged, with office clerks exhibiting higher adherence compared to housekeepers (OR = 3.18, p = .008). Planned (OR = 0.38, p = .025) and unplanned but wanted pregnancies (OR = 0.42, p = .045) showed lower adherence. Regression analysis indicated that Neuroticism (β = −0.21, p = .032) and EOE (β = −0.28, p = .008) negatively impacted adherence.

**Conclusion:**

Specific personality traits, sensitivity, education, occupation, and pregnancy significantly influence adherence. Tailored interventions that enhance conscientiousness, address mental openness and sensitivity, and consider individual socio-demographic context are needed to promote better adherence and improve maternal and fetal health outcomes in high-risk pregnancies.

## Introduction

Medication adherence, the degree to which patients follow prescribed treatments, is essential for treatment efficacy. Unlike “compliance”, adherence emphasizes patient autonomy in decision-making and treatment acceptance.^1^^–^^3^ Non-adherence is a pervasive issue, compromising treatment success, patient wellbeing, and healthcare systems,^4^^–^^8^ as it poses a significant challenge to both public and personal health.^9^ Non-adherence rates range between 20% and 50%, especially in chronic conditions, leading to adverse outcomes and increased costs.^10^ Good adherence reduces mortality and clinical complications, positively impacting quality of life and healthcare costs.^11^^–^^13^

Shared decision-making and addressing patient-specific factors, including knowledge, concerns, and the patient-physician relationship, are crucial for improving adherence. This is particularly important during pregnancy, where maternal and fetal well-being are paramount, especially in high-risk pregnancies.^14^^,^^15^

High-risk pregnancies, with elevated risks of adverse outcomes, can arise from different maternal or fetal conditions.^16^^,^^17^ Non-adherence in pregnancy can lead to complications, increased hospitalizations, higher costs,^18^^,^^19^ and negative impacts on child development.^20^^–^^22^ Women often overestimate medication risks during pregnancy, leading to treatment avoidance.^23^^–^^27^ Despite its importance, research on medication adherence in pregnant women is limited, with reported rates varying between 17% and 56% in those with chronic conditions.^28^

A series of factors may influence adherence during pregnancy, including disease characteristics, patient-physician dynamics, socio-economic context, healthcare system quality, and psychological state.^29^ Maternal concerns about the potential effects of medication on the fetus, even when addressed by healthcare providers, often hinder adherence. In this regard, the availability of evidence-based information and the establishment of empathetic, trust-based therapeutic relationships have been shown to enhance adherence.^13^^,^^30^^,^^31^

While the primary aim of the present study is to examine dispositional and situational factors, it is important to acknowledge that specific clinical conditions may substantially affect adherence behaviors during pregnancy. Chronic conditions such as asthma, inflammatory bowel diseases, mood disorders, substance use disorders (including nicotine dependence), and neurological conditions like epilepsy are commonly linked to lower treatment adherence, particularly during pregnancy.^32^^–^^36^ The persistent nature of these illnesses, combined with the intricacies of their pharmacological management in gestational contexts, often poses significant challenges. Among these, nicotine dependence stands out as a well-established indicator of non-adherence, not only affecting maternal outcomes and therapeutic success, but also potentially confounding the interpretation of psychological variables in studies assessing adherence behavior.^37^^–^^39^ Furthermore, medications belonging to specific pharmacological categories, particularly antidepressants, benzodiazepines, and other anxiolytics, are frequently adjusted, reduced, or discontinued during pregnancy.^40^ This trend is especially evident among women with a prior history of mood or anxiety disorders, where concerns about fetal safety, potential side effects, and stigma often influence treatment decisions and clinical management strategies.^41^ These considerations underscore the multifaceted nature of adherence and the need for integrative models that encompass clinical, pharmacological, and psychological dimensions.

Psychosocial and psychopathological factors also play a role, while impacting adherence and pregnancy outcomes.^42^^–^^45^ Social support, marital satisfaction, emotional stability, and anxiety management are essential for adherence and healthy pregnancy behaviors.

Furthermore, demographic factors such as age, education, and economic status contribute to the overall well-being of pregnant women, influencing physical activity, nutrition, and weight gain.^46^^,^^47^ Pregnant women experiencing depression or anxiety tend to exhibit less healthy habits, negatively affecting pregnancy outcomes.^48^^–^^53^ Personality and temperament likely influence women’s perceptions of medication side effects and teratogenic risk, impacting adherence and outcomes. Personality, shaped by genetic and environmental factors, affects thoughts, feelings, and behaviors.^54^^,^^55^

The Big Five model categorizes personality traits into neuroticism, extraversion, conscientiousness, agreeableness, and openness to experience. Neuroticism, characterized by emotional instability, is linked to poorer well-being and increased healthcare needs, predisposing individuals to depression and anxiety.^56^^–^^59^

Neuroticism is also associated with non-adherence and negative beliefs about medication during pregnancy.^60^^,^^61^ Conversely, conscientiousness, marked by self-regulation and impulse control, correlates with better adherence, especially when combined with perceived therapeutic benefits.^54^^,^^62^^–^^64^

Approximately 15%–20% of the population displays high sensitivity to stimuli, processing information more deeply. This sensory processing sensitivity (SPS) involves increased central nervous system sensitivity and deeper cognitive processing.^65^ SPS includes pausing in new situations, sensitivity to subtle stimuli, and deeper cognitive processing for coping, driven by heightened emotional reactivity. SPS represents individual differences in somatic sensation, reflecting how the brain processes sensory information.^65^^–^^68^

Pregnant women with “high sensitivity” may face challenges maintaining well-being and adhering to therapeutic recommendations. SPS is positively associated with neuroticism, with “highly sensitive persons” experiencing hyperarousal and heightened emotional responses under stress. However, the correlation between SPS and neuroticism is moderate.^65^^,^^69^

Recent psychological and neurobiological research has also highlighted a potential overlap between SPS and Emotional Dysregulation (ED).^70^^,^^71^ ED refers to difficulties in modulating emotional responses, particularly under stress, and has gained attention as a transdiagnostic vulnerability factor in mood and neurodevelopmental disorders.^72^ While distinct, SPS and ED share important features, such as heightened emotional reactivity, sensitivity to environmental cues, and reduced capacity for top-down emotion regulation.^73^ These shared characteristics may be particularly relevant in the context of high-risk pregnancy, where emotional regulation plays a crucial role in treatment adherence and maternal well-being.^74^

Given these premises, this study investigated therapeutic adherence in a sample of Italian women with high-risk pregnancies, exploring the role of personality traits and socio-demographic variables (educational level, civil status, parity, and trimester of pregnancy) in influencing adherence. The potential correlation between neuroticism, sensitivity to stimuli, and therapeutic adherence was also examined.

Furthermore, we should also specify that, within the context of this study, adherence encompasses the extent to which women with high-risk pregnancies follow the medical recommendations provided by their healthcare providers. These recommendations primarily include the correct and timely intake of prescribed medications, but also encompass adherence to behavioral advice such as dietary modifications, adequate rest, and attendance at scheduled medical consultations. We acknowledge that adherence is a complex behavior influenced by multiple factors, and our study focuses on exploring the roles of personality traits and sensory sensitivity in this process.

## Materials and Methods

### Study participants and procedure

This naturalistic case–control study involved a single assessment of pregnant women at the gynecological outpatient service for high-risk pregnancies at Villa Sofia—V. Cervello Hospital, a public healthcare provider in Palermo, Southern Italy.

Pregnant women were evaluated by psychologists trained in the administration of psychometric tests, and data were recorded in a database.

Data collected at entry included individual information that was left anonymous for clinical or other research purposes.

We did not use specific criteria for the inclusion of patients in this database other than their “wish to be interviewed” and having said they “wanted to participate” in a future survey. Each patient could decide whether to accept or decline his/her inclusion in the study. The decision to accept or decline did not in any way affect the care the patient received. The patient could withdraw his/her consent at any time without giving any explanation. This study was conducted according to the WMA Declaration of Helsinki—Ethical Principles for Medical Research Involving Human Subjects and was approved by the Ethics Committee of Palermo 2 (no. 486/2022).

The only inclusion criterion included the “high-risk pregnancy status.”

The National Institutes of Health (NIH) has outlined several broad categories that may create risks during a pregnancy.^75^ These risks may be due to factors in the pre-existing maternal medical conditions (hypertensive disorders, polycystic ovarian syndrome, diabetes, renal disease, autoimmune disease, thyroid disease, infertility, obesity, HIV/AIDS), age (adolescent, first-time pregnancy after 35 years of age), lifestyle factors (alcohol, tobacco, illicit drugs) and condition of pregnancy (multiple gestation, gestational diabetes, preeclampsia and eclampsia). Events that occur during a pregnancy may also lead to high-risk status. Risks may also be classified as biological (genetic, nutritional, general health status, medical, or obstetric disorders), psychological (maternal behaviors, lifestyle, emotional disorders, disturbed interpersonal relationships, inadequate social support, unsafe cultural practices), socio-demographic (lack prenatal care, insurance status, low income, marital status, race, ethnicity), or environmental factors (hazards in workplace and general environment, chemicals, gases, radiation).

The criteria of exclusion were limited to the impossibility of giving informed consent.

A total of 90 women (mean age and SD: 30.4 ± 5.0 years), recruited between March 2022 and June 2023, were included in the present study. The socio-demographic characteristics of the sample were listed in Table 1.

**Table 1.** Socio-demographics features of the sample

	*n* (%)
Age ≤30 yrs >30 yrs	41 (45.6%) 49 (54.4%)
Trimester First Second	41 (45.6%) 49 (54.4%)
Civil status Married Cohabitant	55 (61.1%) 35 (38.9%)
Parity Primiparous Pluriparous	33 (36.7%) 57 (63.3%)
Educational level Middle school High school Graduate	53 (58.9%) 27 (30.0%) 10 (11.1%)
Work Housekeeper Student Office clerk Manager Freelancer Teacher Other	72 (80.0%) 2 (2.2%) 8 (8.9%) 1 (1.1%) 1 (1.1%) 2 (2.2%) 4 (4.4%)
Pregnancy Planned Unplanned but wanted Unplanned and unwanted	53 (58.9%) 32 (35.6%) 5 (5.6%)
Reason for accessing “high risk pregnancy” outward Mother illnesses Fetus illnesses Placental or pregnancy pathologies Fetus and mother illnesses	52 (57.8%) 10 (11.1%) 3 (3.3%) 25 (27.8%)

All subjects were first assessed by a clinical evaluation with the ensuing diagnoses.

After a complete description of the study, a written informed consent was obtained from each subject to participate in the study.

### Assessment scales

#### Personality inventory

The Personality Inventory (PI) is a 20-item self-report questionnaire that evaluates personality factors according to the Big Five model.^76^

The questionnaire has five sub-scales, each of which investigates *Extraversion* defined by the search for aggregation, assertiveness, positive emotionality, the search for excitement; *Conscientiousness* referring to a sense of duty and self-discipline; *Neuroticism* understood as a tendency to emotional instability; *Mental Openness* in the sense of openness to experiences and intellectual curiosity, and *Friendliness* understood as trust in others and the ability to cooperate. Each item was scored on a 5-point scale, from 1 = strongly disagree to 5 = strongly agree.

#### Highly sensitive person scale

The *Highly Sensitive Person* (HSP) Scale is a tool that measures Sensory Processing Sensitivity (SPS), a personality trait characterized by greater depth of information processing, greater emotional reactivity and empathy, greater awareness of environmental details, and ease of overstimulation.^77^^–^^79^ The HSP Scale is a questionnaire composed of 12 items, self-report questions with positive and negative cognitive and emotional responses to various environmental stimuli. It is composed of three subscales: (1) *Low sensory threshold (LST)*, that is sensitivity to subtle external stimuli; (2) *Ease of excitement (EOE)*, that is being easily overwhelmed by internal and external stimuli; (3) *Aesthetic Sensitivity (AES)*, that is openness to, and enjoyment of, aesthetic experiences and positive stimuli. The possible range of scores is 4–28, where a score of 4–12 indicates low sensitivity, a score of 13–20 indicates medium sensitivity, and a score ˃21 indicates high sensitivity. The psychometric properties and validity of the 27-item HSP scale, as well as shorter versions,^80^^–^^83^ have been validated in multiple studies.

#### Morisky Medication Adherence Scale

The *Morisky Medication Adherence Scale* (MMAS-8) is an 8-item self-report measure widely used across various cultures to assess medication-taking behavior.^84^ To provide a clearer understanding of the assessment, some examples of questions include: “Do you ever forget to take your medicine?” and “When you travel, do you forget to bring your medicine with you?.” The first seven items are dichotomous, with answer categories of “yes” or “no”, while the last item is a five-point Likert scale question.

Compared to the original Morisky scale, it has the following characteristics: the inclusion of four items aims to identify and individuate the circumstances and/or situations related to adherent behavior (adherence to treatment) (adherent behavior); the questions are worded to avoid an “always say yes” bias (i.e., the wording of item 5 is reversed to prevent the tendency to answer a series of questions in the same way regardless of their content).

Each “no” answer is scored as 1, and each “yes” answer is scored as 0, except in step 5, where each “yes” answer is scored as 1 and each “no” answer is scored as 0. For item 8, the code (0–4) should be standardized by dividing the result by 4 to calculate a summed score.

Total scores on the MMAS-8 range from 0 to 8, with scores of 8 reflecting high adherence, 7 or 6 reflecting medium adherence, and <6 reflecting low adherence. Morisky and its derivatives have moderate to high reliability and criterion validity in some studies, but there is still room for improvement in translational validity, including content validity. Consequently, clinicians and researchers should be cautious before using them as measurements and should consider two key points: (1) Whether the MMAS is appropriate to use to achieve the goal of the study or intervention. (2) Whether the MMAS has been validated in this specific situation, which may be different from the original validation environment. MMAS-4 and MMAS-8 were designed to describe patients’ medication-taking behavior, but they do not appear to be able to comprehensively assess the reasons for or predictors of medication adherence. They may be considered a good estimate of medication-taking behavior, but they are not good explanatory tools for understanding why patients are non-adherent, which may lead to a poor relationship between the Morisky scale and objective measures of clinical outcome. In addition, they are good screening and monitoring tools for identifying patients who may have medication adherence problems.

#### Assessment of pregnancy planning

To assess pregnancy planning, participants were categorized into one of three groups based on self-report data collected during the initial interview. These categories were designed to understand the participants’ perspectives on their pregnancy planning experiences:

- *Planned Pregnancy:* Defined as a pregnancy that was actively intended and desired by the woman and, if applicable, her partner, at the time of conception.
- *Unplanned but Wanted Pregnancy:* Defined as a pregnancy that was not actively intended at the time of conception but was welcomed and desired upon discovery.
- *Unplanned and Unwanted Pregnancy:* Defined as a pregnancy that was neither intended nor desired at the time of conception or following confirmation.

During the initial interview, participants were asked questions to understand their experiences related to pregnancy planning. To ensure sensitivity, the questions were framed to be as neutral and non-judgmental as possible. Examples of questions included: “Thinking back to the time before you became pregnant, were you and your partner actively trying to conceive?”, “When you found out you were pregnant, what were your initial feelings about the pregnancy?”, and “At that time, did you feel that becoming pregnant was something you wanted in your life?.” Participant responses to these questions were used to categorize them into the appropriate pregnancy planning group.

### Data analysis

Descriptive statistics were calculated for all demographic and clinical variables. Continuous variables were presented as mean ± SD, range (min–max), or median, as appropriate (see Table 1 for details on mother’s age, pregnancy trimester, civil status, parity, educational qualification, work, type of pregnancy, and reason for accessing high-risk pregnancy outward services). Categorical variables were summarized as frequencies and percentages.

Normality of distribution was assessed using the Kolmogorov–Smirnov test. Group comparisons for continuous variables were conducted using independent-samples *t*-tests (for two groups) and one-way ANOVA (for more than two groups). Chi-square tests were employed for analyzing categorical variables. Non-parametric tests, specifically the Mann–Whitney *U* test and Kruskal–Wallis test, were used when data did not meet normality assumptions. Relationships between study variables (neuroticism, maternal adherence, low sensory threshold, ease of excitement, aesthetic sensitivity, total high sensitivity, etc.) were examined using Pearson (for parametric data) or Spearman rank (for non-parametric data) correlations.

Given that the Morisky Medication Adherence Scale generates ordinal data and adherence scores are often non-normally distributed, ordinal logistic regression was employed. This type of regression analysis is suitable for predicting an ordinal outcome variable based on a set of predictor variables, without requiring the assumption of normality. It models the odds of being in a higher adherence category based on the predictors. The proportional odds assumption, a key requirement for ordinal logistic regression, was tested and not violated.

A major limitation of this study is the small sample size, which increases the risk of both Type I and Type II errors. Therefore, these results should be considered preliminary. A *p*-value <0.05 was considered statistically significant. All analyses were performed by using SPSS 27.0.^85^

## Results

### Assessment scales and clinical characteristics

Table 2 summarizes the key values obtained from the rating scales administered to the study sample.

**Table 2.** Assessment scale scores, subdivided by each domain

Assessment scale	Min	Max	Mean value	Standard deviation
Highly Sensitive Person Scale				
Aesthetic Sensitivity (AES)	7	28	18.20	4.57
Low Sensory Threshold (LST)	6	25	13.87	4.59
Ease of excitement (EOE)	4	28	15.37	6.10
HSP total score	24	72	47.43	10.57
Morisky Medication Adherence Scale	2	7	5.75	1.45
Personality inventory				
Neuroticism	4	25	9.07	2.52
Conscientiousness	8	19	13.82	2.51
Mental Openness	7	17	11.96	2.35
Extraversion	9	20	14.09	2.12
Friendliness	4	17	12.30	2.62

The PI yielded average scores across its subscales, although specific cut-off values for comparison were unavailable.

Regarding sensory processing sensitivity, the HSP scale indicated moderate levels within the sample. Participants exhibited a moderate tendency toward aesthetic sensitivity, captured by the AES dimension (mean = 18.20); a moderate sensitivity to external stimuli, reflected in the EOS dimension (mean = 13.87); and a moderate susceptibility to being overwhelmed by stimuli, as measured by the LST dimension (mean = 15.37). The overall HSP score (mean = 47.43) further corroborated a moderate level of sensory processing sensitivity.

In contrast, the MMAS revealed low adherence among the participants (mean = 5.75), suggesting that, on average, they experienced challenges in consistently adhering to their prescribed medication regimen.

### Correlational and comparative analyses

Before proceeding to the correlation and comparison analyses of the variables considered in the study, we applied the Kolmogorov–Smirnov test.

Table 3 contains the Kolmogorov–Smirnov test values for all variables. With the exception of the “LST” dimension, all variables had a *p* value <0.05, leading us to reject the null hypothesis that the variables have a normal distribution. Therefore, non-parametric tests were used for subsequent group comparisons, while correlation analyses were performed to explore relationships between variables.

**Table 3.** Normality distribution analysis with Kolmogorov-Smirnov test

	Kolmogorov-Smirnov^a^		
	Stats.	Df	*p*
Highly sensitive person scale			
Aesthetic Sensitivity (AES)	.108	90	0.011
Low Sensory Threshold (LST)	.089	90	0.075
Ease of excitement (EOE)	.130	90	0.001
HSP total score	.110	90	0.009
Morisky Medication Adherence Scale	.228	90	< 0.001
Personality inventory			
Neuroticism	.116	90	0.004
Conscientiousness	.117	90	0.004
Mental openness	.119	90	0.003
Extraversion	.141	90	< 0.001
Friendliness	.132	90	0.001

These analyses reveal a complex interplay between personality traits, sensory processing sensitivity, and medication adherence. Key correlations and group differences are presented in Table 4.

**Table 4.** Correlations between assessment scale scores using Spearman-rank correlation (only statistically significant values were included)

		*ρ*	*p*
Neuroticism	Low Sensory Threshold (LST)	0.409	< 0.001
	Ease of excitement (EOE)	0.471	< 0.001
	HSP total score	0.416	< 0.001
	Morisky Medication Adherence Scale	−0.258	0.014
Conscientiousness	Aesthetic Sensitivity (AES)	0.214	0.043
	HSP total score	0.228	0.030
	Mental openness	0.379	< 0.001
Mental openness	Aesthetic Sensitivity (AES)	0.447	< 0.001
Aesthetic Sensitivity (AES)	Low Sensory Threshold (LST)	0.262	0.013
Low Sensory Threshold (LST)	Ease of excitement (EOE)	0.453	< 0.001
Ease of excitement (EOE)	Morisky Medication Adherence Scale	−0.312	0.003

Neuroticism showed a strong positive association with several aspects of sensory processing sensitivity. Higher neuroticism scores were linked to increased sensitivity to subtle stimuli (LST, *Rho* = 0.409, *p* < 0.001), a greater tendency to be overwhelmed by stimuli (EOE, *Rho* = 0.471, *p* < 0.001), and a higher overall sensitivity (HSP Total Score, *Rho* = 0.416, *p* < 0.001). Conversely, neuroticism was negatively correlated with medication adherence (MMAS, *Rho* = −0.258, *p* = 0.014).

Conscientiousness was positively linked to AES (*Rho* = 0.214, *p* = 0.043), HSP Total Score (*Rho* = 0.228, *p* = 0.030), and mental openness (*Rho* = 0.379, *p* < 0.001). Moreover, mental openness itself was also positively correlated with AES (*Rho* = −0.447, *p* < 0.001).

Interestingly, the subscales within the HSP Scale also showed intercorrelations. AES was positively correlated with LST (*Rho* = 0.262, *p* = 0.013), while a negative correlation emerged between LST and EOE (*Rho* = −0.453, *p* < 0.001).

Finally, EOE was negatively correlated with MMAS score (*Rho* = −0.312, *p* = 0.003).

For comparisons between two independent groups, either the Mann–Whitney *U* test or Student’s *t*-test was used, depending on whether the data met the assumptions of normality (Table 5a). For comparisons between three or more groups, the Kruskal-Wallis test was employed (Table 5b). Several statistically significant differences emerged and are listed below.

**Table 5a.** Intergroup comparisons using Student’s t-test or Mann-Whitney test based on normality distribution of each variable (only statistically significant values were included)

Groups	Scale scores	Z	*p*
Age (≤30 years vs. >30 years)	Friendliness	−2.138	0.032
Trimester (first vs. second)	Low Sensory Threshold (LST)	−2.587 (*t*)	0.011*
Civil status (married vs. cohabitant)	Low Sensory Threshold (LST)	−2.247 (*t*)	0.027
	HSP total score	−2.130	0.033

**Table 5b.** Intergroup comparisons using Kruskal-Wallis test (only statistically significant values were included)

Groups	Scale scores	H	*p*
Educational level			
	Aesthetic sensitivity (AES)	9.622	0.008
	Middle school vs. graduate	7.914	0.015*
Work			
	Neuroticism	12.611	0.049
	Office clerk vs. teacher	10.000	0.033*
	Office clerk vs. student	10.000	0.033*
	Office clerk vs. others	12.000	0.011*
Pregnancy type			
	Aesthetic Sensitivity (AES)	9.160	0.010
	Unplanned but wanted vs. unplanned and unwanted	6.801	0.027*
	Morisky Medication Adherence Scale	12.011	0.002
	Unplanned but wanted vs. planned	6.517	0.032*
	Unplanned but wanted vs. Unplanned and unwanted	9.498	0.006*
	Mental openness	7.835	0.020
	Unplanned but wanted vs. planned	6.517	0.032*
Reason for accessing			
	Ease of excitement (EOE)	13.931	0.003
	Fetus and mother illnesses vs. mother illnesses	8.579	0.020*

Younger women (≤30 years) scored significantly higher on Friendliness compared to older women (>30 years) (*Z* = −2.138, *p* = 0.032). Women in their first trimester of pregnancy had significantly higher LST scores (*t* = −2.587, *p* = .011). Married women showed significantly higher LST scores (*t* = −2.247, *p* = 0.027) and total HSP scores (*Z* = −2.130, *p* = 0.033) compared to unmarried women. Women with a middle school education had significantly higher AES (*H* = 7.914, *p* = 0.015) compared to those with graduate degrees. Women working as office clerks demonstrated lower Neuroticism scores compared to teachers (*H* = 10,000, *p* = 0.033), students (*H* = 10,000, *p* = 0.033), and those in “other” occupations (*H* = 12,000, *p* = 0.011). Women with unplanned and unwanted pregnancies had significantly higher AES scores (*H* = 6.801, *p* = 0.027) and MMAS scores (*H* = 9.498, *p* = 0.006) compared to those with unplanned but wanted pregnancies. Additionally, women with planned pregnancies had higher MMAS scores (*H* = 6.517, *p* = 0.032) and Mental Openness scores (*H* = 6.517, *p* = 0.032).

Women categorized as high-risk due to fetus and mother illnesses had significantly lower EOE scores (*H* = 8.579, *p* = 0.020).

### Ordinal logistic regression

Medication adherence, as measured by the 8-item MMAS, was assessed for normality using the Kolmogorov–Smirnov test, which indicated a non-normal distribution (*p* < .001). The MMAS-8 generates ordinal data, representing distinct levels of adherence, rather than continuous data suitable for parametric analysis. Moreover, adherence scores frequently exhibit a skewed distribution, with a tendency towards higher reported adherence levels.

Therefore, ordinal logistic regression, as indicated in the “Data analysis” paragraph, was employed to assess the factors associated with medication adherence. This approach allows us to model the odds of being in a higher adherence category based on the predictor variables, without assuming normality. The proportional odds assumption, a requirement for ordinal logistic regression, was tested using the Test of Parallel Lines and was not violated (*χ*^2^ = 341.49, *p* = .618).

The model included several predictor variables: PI subscales, HSP subscales, age, trimester, civil status, parity, educational level, work status, pregnancy types, and reason for accessing high-risk pregnancy outward services.

The model was statistically significant (*χ*^2^ = 53.66, *p* < .001), indicating that the included predictors significantly improved the prediction of medication adherence compared to a model with no predictors. Several personality traits were significantly associated with adherence. Higher mental openness (*β* = −0.41, *p* < .001) and higher EOE scores (*β* = −0.22, *p* < .001) were associated with *lower* medication adherence. Conversely, higher conscientiousness (*β* = 0.25, *p* = .018) was associated with *higher* adherence. Higher educational levels (high school: *β* = 2.77, *p* = .004; graduate: *β* = 2.44, *p* = .007) were also associated with increased adherence compared to the lowest educational level (middle school).

Office clerks (OR = 2.25, *p* = .039, 95% CI [1.03, 6.06]), freelancers (OR = 5.00, *p* = .018, 95% CI [1.29, 13.70]), and teachers (OR = 5.06, *p* = .002, 95% CI [2.81, 12.26]) demonstrated significantly higher medication adherence compared to housekeepers in an ordinal logistic regression model. These odds ratios indicate the increased likelihood of these professions reporting higher adherence compared to housekeepers. The 95% confidence intervals provide the range of plausible values for these odds ratios.

Planned pregnancies (*β* = −3.02, *p* = .010) and unplanned but wanted pregnancies (*β* = −3.85, *p* = .002) were associated with lower adherence compared to unplanned and unwanted pregnancies.

Pseudo R-squared values (Cox and Snell R^2^ = 0.45, Nagelkerke R^2^ = 0.46, McFadden R^2^ = 0.14) indicated that the model explained a moderate proportion of the variance in medication adherence. Goodness-of-fit tests (Pearson χ^2^ = 1303.12, *df* = 1085, *p* < .001; Deviance χ^2^ = 341.49, *df* = 1085, *p* < .001) suggested some deviation from perfect fit, indicating that there may be other unmeasured factors influencing adherence (Table 6).

**Table 6.** Ordinal Logistic Regression Results for Factors Associated with Medication Adherence (Measured by the 8-item Morisky Medication Adherence Scale)

Predictor variable	Coefficient (β) or odds ratio	*p* value	95% CI (where applicable)	Interpretation
Highly Sensitive Person Scale				
Ease of excitement	−0.22	< .001		Higher ease of excitement is associated with lower adherence
Aesthetic sensitivity				Not significant in the model
*Personality inventory*				
Mental openness	−0.41	< .001		Higher mental openness is associated with lower adherence
Conscientiousness	0.25	.018		Higher conscientiousness is associated with higher adherence
*Educational level*				
High school (vs. middle school)	2.77	.004		Higher adherence
Graduate (vs. middle school)	2.44	.007		Higher adherence
*Work*				
Office clerk (vs. housekeeper)	OR = 2.25	.039	[1.03, 6.06]	Higher adherence
Freelancer (vs. housekeeper)	OR = 5.00	.018	[1.29, 13.70]	Higher adherence
Teacher (vs. housekeeper)	OR = 5.06	.002	[2.81, 12.26]	Higher adherence
*Pregnancy type*				
Planned (vs. unplanned and unwanted)	−3.02	.010		Lower adherence
Unplanned but wanted (vs. unplanned and unwanted)	−3.85	.002		Lower adherence

## Discussion

This study investigated medication adherence in Italian women with high-risk pregnancies and explored the influence of personality traits on treatment compliance. The sample exhibited low adherence (MMAS average = 5.75), echoing existing literature highlighting the pervasive challenge of non-adherence, particularly during pregnancy, despite its recognized impact on maternal and fetal health outcomes.^86^^–^^90^

Building on the observed low adherence rates in our sample, this paragraph delves into the intricate relationship between personality traits and medication adherence during pregnancy. We aim to explore how individual differences in personality may either facilitate or impair adherence to prescribed treatment regimens.

The observed positive association between *conscientiousness* and medication adherence aligns with existing research.^91^ Conscientiousness, a personality trait encompassing organization, discipline, and a strong sense of duty, appears to promote adherence. This is likely because conscientious individuals are typically methodical and planful, with a strong self-discipline and inclination to follow rules, which helps them to integrate medication schedules into their routines.^92^^–^^94^ This effect may be particularly pronounced in younger individuals,^91^ potentially because they are still developing consistent health.^95^^,^^96^

Lower medication adherence in pregnant women correlates with higher *neuroticism* scores, a finding supported by existing research,^97^^,^^98^ although regression analysis might not always identify neuroticism as a significant overall predictor of medication adherence. Several pathways can explain how higher neuroticism contributes to non-adherence. Heightened anxiety and negative emotions, characteristic of this trait, can increase stress reactivity, thereby reducing confidence in handling challenging situations. This affects how individuals manage difficulties, leading to worry and altered perceptions.^99^^–^^101^ High neuroticism also involves psychological pressure, unrealistic thoughts, and depressive feelings. Individuals may cope with these feelings by adopting maladaptive strategies like experiential avoidance, prioritizing control. However, emotional regulation and cognitive flexibility may offer more effective coping mechanisms, potentially alleviating depressive symptoms.^102^^–^^105^ Neuroticism may also indirectly affect adherence by reducing perceived social support and increasing focus on treatment downsides.^100^^,^^106^^–^^114^ Self-medication with antidepressants and anxiolytics among neurotic women can further complicate treatment adherence during pregnancy and impact maternal and fetal health.^115^ Maternal neuroticism can also negatively influence birth outcomes by affecting self-care, childcare, and physiological stress responses.^116^^–^^118^ High neuroticism scores correlate with increased hypothalamic–pituitary-adrenocortical axis (HPA) and sympathetic nervous system (SNS) reactivity to stress, which during pregnancy, can affect labor and potentially increase interventions and complications.^119^^–^^123^

At variance with our expectations, heightened mental openness correlated with diminished adherence: this is in contrast with the conventional understanding that open individuals readily embrace new information and health recommendations.^124^ This finding warrants further investigation to clarify the underlying mechanisms. One possibility lies in pregnancy’s unique context: greater openness may lead to broader information-seeking, potentially exposing individuals to concerns about medication risks, which, when coupled with higher neuroticism, could amplify anxiety and contribute to non-adherence.^125^^,^^126^ Moreover, the tendency to question norms, characteristic of open individuals, may result in less reliance on medical advice.^127^ This independent mindset, coupled with the emotional intensity of pregnancy and exposure to diverse, sometimes conflicting, information from external sources, could lead individuals to make autonomous decisions about medication different from prescribed regimens.^124^^,^^128^

We also explored the connection between sensitivity to stimuli and medical adherence in high-risk pregnant women, focusing on *SPS* (a trait found in 10%–20% of the population involving heightened awareness and reactivity to stimuli, leading to deeper information processing and its impact).^65^^,^^129^ While SPS can foster empathy and creativity, its high reactivity may also present challenges, particularly for Highly Sensitive Persons.^130^^–^^136^ Heightened sensitivity, or *EOE*, can predict poorer medication adherence, potentially due to cognitive overload and stress.^137^^,^^138^ Although a low sensory threshold was not directly linked to adherence in the regression model, the observed higher *LST* in the first trimester could indirectly impact adherence through increased stress and disrupted routines, maybe due to hormonal fluctuations and physiological adaptations, such as changes in the HPA and elevated cortisol levels.^139^^,^^140^ This overreaction to stimuli may stem from difficulty managing sensations and emotions, potentially linking sensitivity to traits like neuroticism, characterized by ED. The neurobiological basis of these traits may involve altered emotional processing and rational control. Specifically, functional Magnetic Resonance Imaging (fMRI) studies show that neuroticism is negatively correlated with activation in brain regions like the dorsomedial prefrontal cortex (dmPFC), middle frontal cortex, and inferior frontal cortex during emotion regulation and cognitive reappraisal.^141^^–^^150^ This reduced activation may impair negative emotion downregulation, cognitive control, and self-monitoring. Furthermore, neuroticism is linked to decreased connectivity between the amygdala and dmPFC, suggesting a reduced cognitive control over emotions.^151^^,^^152^

Therefore, HSPs may also exhibit functional alterations in these brain regions, impairing top-down emotional regulation: they rationalize events but struggle to manage emotions and subsequent reactions.

The current findings further support the hypothesis that individuals with high SPS may be more vulnerable to ED processes, particularly under conditions of heightened physiological and psychological stress. This vulnerability may reflect not only psychological reactivity but also underlying neurobiological sensitivity. Neurogenetic research has linked SPS to specific polymorphisms in genes involved in serotonin (5-HTTLPR), dopamine (DAT1, DRD4), and norepinephrine (ADRA2b) pathways, all of which modulate emotional responsivity and perceptual sensitivity.^153^^–^^155^ These findings suggest that high SPS individuals may exhibit amplified responses to environmental and emotional stimuli, which could contribute to difficulties in emotion regulation. In this framework, emotion regulation difficulties have been identified as a transdiagnostic mechanism linking early sensitivity traits to increased risk for psychopathology.^70^^,^^71^ Importantly, in perinatal populations, ED has been shown to compromise treatment adherence by increasing emotional avoidance, distress intolerance, and dropout risk.^74^^,^^156^ These interconnected dimensions offer a plausible explanation for the negative influence of both SPS (especially EOE) and neuroticism on medication adherence observed in our sample.

In our study, we observed several additional findings that, while not central to our primary hypotheses regarding personality and adherence, warrant mention.

We found that certain professional roles, such as *office clerks*, correlated with diminished levels of neuroticism, and that individuals in these occupations, along with *teachers* and *freelancers*, tended to exhibit greater compliance with prescribed medical treatments compared to *housekeepers.* However, the relationship is not always straightforward. Interestingly, women employed as office clerks demonstrated higher neuroticism than other occupational groups, yet this did not negatively impact their adherence, suggesting that other occupation-related factors, such as work schedule flexibility and access to healthcare resources, may be more influential. This suggests a complex relationship between occupation, personality, and health behaviors, where factors like stress levels and autonomy may play a mediating role. Further research is needed to fully understand these dynamics and inform personalized strategies for enhancing medication adherence.

Our analysis also revealed a positive correlation between higher education levels and improved adherence. This likely stems from increased health literacy, better understanding of treatment rationale, and greater ability to navigate the healthcare system. This reinforces the need for clear communication and accessible health information for all patients.

A higher medication adherence was also revealed among women with *unplanned and unwanted* pregnancies compared to those with *planned* or *unplanned but wanted* pregnancies. This unexpected finding requires to be deepened, while considering potential differences in motivation, access to care, or other psychosocial factors.^157^^,^^158^ This discrepancy highlights the complex and context-dependent nature of adherence behaviors in this population. The complexity of this issue is highlighted by the observation that women with *unplanned but wanted* pregnancies may demonstrate higher adherence than those with planned pregnancies. One hypothesis is that women with unplanned but wanted pregnancies, feeling less prepared for childbirth, may rely more on healthcare providers’ recommendations. This could also explain their higher degree of mental openness, a trait associated with curiosity, adaptability, and acceptance of new experiences. This openness might make them more receptive to lifestyle changes and therapeutic advice. However, it is crucial to acknowledge the potential negative effects of unplanned pregnancies that are associated with increased risks of obstetric complications, delayed antenatal care, prenatal and postnatal depression, relationship difficulties, and poorer health outcomes for children.^157^^,^^158^

Finally, our findings indicate that married women demonstrate higher LST and overall SPS. Although these factors were not directly linked to medication adherence in our regression analysis, they raise questions about the potential interplay between social support, marital status, and sensitivity in influencing health behaviors.

### Limitations

This study has several limitations that warrant consideration. The cross-sectional design precludes establishing causal relationships between the examined variables. While the study identifies associations between personality traits, sensory processing sensitivity, socio-demographics, and adherence, it cannot determine whether these factors directly cause changes in adherence behavior. In particular, the study’s occupational findings, specifically the observed differences in adherence between housekeepers and other professions, may be influenced by variations in educational level across these groups, a factor not directly addressed in the current analysis.

The reliance on self-reported measures of both adherence and psychological constructs introduces potential biases. Participants may over-report adherence due to social desirability or recall difficulties. Similarly, self-reported personality and sensitivity measures are susceptible to response bias and may not accurately reflect underlying constructs.

Another important limitation of the present study is the absence of data regarding the participants’ specific medical diagnoses and the types of pharmacological treatments prescribed or discontinued during pregnancy. Although our primary objective was to examine psychological and socio-demographic predictors of adherence, we acknowledge that clinical conditions, such as chronic illnesses or psychiatric disorders, and the pharmacological agents involved (e.g., antidepressants, anxiolytics, or anti-epileptic drugs) may substantially influence adherence behavior and potentially act as confounding variables. The lack of such information precluded their inclusion in our statistical models. Future studies should consider integrating detailed medical and pharmacological data to provide a more comprehensive and clinically nuanced understanding of adherence patterns in high-risk pregnancies.

The sample, drawn from a single hospital in Palermo, limits the generalizability of the findings to other populations or healthcare settings. The sample size, while adequate for the analyses conducted, may limit the power to detect smaller effects or interactions between variables. The study’s focus on high-risk pregnancies, while clinically relevant, further restricts generalizability to lower-risk pregnancies. Finally, the model, while explaining a moderate amount of variance, leaves a substantial portion unexplained, suggesting the influence of unmeasured factors, such as social support, access to healthcare, or specific pregnancy complications, which could confound the observed relationships. Future research employing longitudinal designs, objective adherence measures, and more diverse samples is needed to address these limitations and should specifically explore the potential mediating or moderating role of educational level in the relationship between occupation and adherence, to provide a more comprehensive understanding of medication adherence in pregnancy.

## Conclusion

Adherence to treatments during pregnancy is critical for both maternal and fetal well-being, directly impacting treatment outcomes and preventing adverse events. Non-adherence arises from a complex interplay of factors, including temperament, socio-demographic influences, and concerns regarding potential drug effects on the fetus. Successfully addressing these challenges necessitates a comprehensive, multifaceted approach that considers the patient, provider, and health system, along with their interactions. Routinely assessing personality traits can help identify individuals at higher risk of non-adherence, enabling targeted interventions. For example, understanding the heightened sensitivity of some individuals can inform communication strategies and support systems. Furthermore, addressing emotional dysregulation, a key aspect of neuroticism and reactivity, may significantly improve adherence. Accessible health information and robust educational interventions are also crucial, and further research is warranted to explore adherence differences across various pregnancy contexts. Integrating personality considerations into adherence models can facilitate more effective, personalized interventions. Multidisciplinary healthcare teams, effective communication, and a patient-centered approach are essential for optimizing adherence and enhancing maternal and fetal well-being.

In particular, future studies should further explore the role of emotional dysregulation as a possible underlying mechanism linking neuroticism and sensory processing sensitivity to suboptimal treatment adherence. Recognizing and addressing ED may improve the precision of interventions designed for highly sensitive or emotionally reactive patients, especially in the context of high-risk pregnancy.

Continued research into these intricate relationships is vital for developing targeted interventions and promoting optimal health outcomes.
